# Depletion of SENP1-mediated PPARγ SUMOylation exaggerates intermittent hypoxia-induced cognitive decline by aggravating microglia-mediated neuroinflammation

**DOI:** 10.18632/aging.203084

**Published:** 2021-05-25

**Authors:** Hongwei Wang, Wei Xiong, Sitong Hang, Yanmin Wang, Sisen Zhang, Song Liu

**Affiliations:** 1 The Second School of Clinical Medicine, Southern Medical University, Guangzhou 510515, Guangdong, China; 2 Department of Respiratory Medicine, Xinhua Hospital, School of Medicine, Shanghai Jiao Tong University, Shanghai 200092, China; 3 China Medical University, Shenyang 110122, Liaoning, China; 4 Affiliated Zhengzhou People's Hospital, The Second School of Clinical Medicine, Southern Medical University, Zhengzhou 450003, Henan, China

**Keywords:** intermittent hypoxia, neuroinflammation, SUMOylation, PPARγ, cognitive dysfunction

## Abstract

Intermittent hypoxia (IH)-associated cognition decline is related to the neuroinflammation of microglia. SUMOylation is a post-translational modification related to multiple human diseases, which can be reversed by SENP1. Studies showed that SENP1 and PPARγ play essential roles in restricting inflammation by blocking NF-κB activation. However, the mechanism remains unclear. Herein, we investigated the precise mechanism underlying SENP1 and PPARγ in cognitive decline after IH insult. Biochemical analysis results revealed that IH triggered the inflammatory response and neuronal apoptosis, increased the SUMOylation of PPARγ, and decreased the level of PPARγ compared to that in the normoxia group. After SENP1 downregulation, the inflammatory response, neuronal apoptosis and the SUMOylation of PPARγ were enhanced, and the level of PPARγ was further decreased *in vitro* and *in vivo*. However, the application of PPARγ agonist, GW1929, abolished the enhancement of inflammation and neuronal apoptosis *in vitro*. The Morris Water Maze results showed that both IH groups mice exhibited longer latency and shorter dwell-time in the goal quadrant than normoxia groups. Notably, SENP1 downregulation aggravated these alterations. Overall, these results showed that SENP1 played an essential role in IH-associated cognitive dysfunction. SENP1 depletion aggravated neuroinflammation and neuronal apoptosis via promoting the SUMOylation of PPARγ, reducing the level of PPARγ, thus exaggerating IH-induced cognitive decline.

## INTRODUCTION

Obstructive sleep apnea (OSA) is a common sleep disorder characterized by repetitive collapse of the upper airway during sleep, which is associated with neurocognitive impairment [1]. Intermittent hypoxia (IH) induced by OSA causes neuronal damage in hippocampus, resulting in cognitive decline [2, 3], and the chronic inflammation of microglia induced by IH plays a crucial role in OSA-related cognitive dysfunction [4–6]. Nuclear factor kappa B (NF-κB) pathway is generally involved in inflammation, including pancreatitis [7], cerebral ischemia [8], and chronic obstructive pulmonary disease [9]. Evidence has shown that peroxisome proliferator-activated receptor γ (PPARγ) can inhibit the expression of inflammatory gene by directly regulating the activity of NF-κB subunits and p38 mitogen-activated protein kinase [10]. Besides, a previous study has reported that SENP1 restricts inflammatory response in adipocytes by promoting the de-SUMOylation of NEMO, inhibiting NF-κB activity and the generation of cytokines [11]. Moreover, our previous study also found that SENP1 inhibited inflammatory response in BV-2 microglial cells with IH treatment [12]. In addition, related study has been indicated that the mechanism underlying the neuroinflammation in IH impairment and the resultant cognitive decline may be associated with the activation of PPARγ [13].

Hippocampus is responsible for learning and memory. Neuroinflammatory response in hippocampus has been confirmed to be related to cognitive dysfunction of many neurodegenerative diseases [14, 15]. Studies showed that proinflammatory cytokines released from microglia in hippocampus could be mediated by PPARγ [16, 17]. PPARγ is a ligand-activated transcription factor of nuclear receptor superfamily, which is expressed in several kinds of cell in tissues, and plays a vital role in inflammatory responses of kidney, intestine, and central nervous system (CNS) [18]. PPARγ and its activators are essential modulators with anti-inflammatory property, which participates in the regulation of NF-κB activity. Once activated, PPARγ is transferred from cell nucleus into the cytosol to interact with NF-κB, resulting in the reduction in the expression of proinflammatory genes such as tumor necrosis factor-a (TNF-a), interleukin-1β (IL-1β), etc. [19]. However, the regulatory mechanism of PPARγ to inflammatory response in IH impairment remains mostly unclear.

SUMOylation is a dynamic post-translational modification process, characterized by the addition or detachment of small ubiquitin-like modifier (SUMO) proteins to lysine residues on target proteins, altering their subcellular localization, activity, and stability [20], which can be reversed by a family of SUMO-specific proteases (SENPs). Among the six SENPs (SENP1~3 and SENP5~7), SENP1 is widely located in cell nucleus and plays an essential role in various kinds of human cancers [21–23]. Accumulating pieces of evidence have demonstrated that SENP1 can deconjugate several SUMOylated proteins, including HIF-1α [24], UBE2T [25], and Akt [26]. Additional evidence has shown that SUMOylation participates in the regulation of PPARγ to the activity of NF-κB and the transcriptional activation of inflammatory response genes [27]. In our previous study, we examined the six SENPs in microglia in response to IH, only the expression of SENP1 showed a significant reduction. Simultaneously, SENP1 overexpression attenuated these effects of apoptosis, nitric oxide synthase expression, and nitric oxide generation in microglia with IH treatment [28]. However, the mechanism of SUMOylation or de-SUMOylation of PPARγ in IH-induced inflammatory response is still unknown. Based on these findings, we hypothesize that SENP1 regulates microglia-mediated inflammatory response toward IH-induced cognitive decline through the de-SUMOylation of PPARγ. In this study, we aimed to determine whether SENP1 depletion would deteriorate cognitive decline in IH mouse models, and if so, by what mechanism. We focused on the regulatory mechanism of SUMOylated or de-SUMOylated PPARγ in the IH-induced inflammatory response of microglia, which has been implicated in the pathophysiology of cognitive dysfunction.

## MATERIALS AND METHODS

### Reagents

GW1929, PPARγ agonist (HY-15655, MedChemExpress), was prepared in Dimethyl sulfoxide (DMSO) at a concentration of 10μM and was added to medium with a final concentration of 0.1% DMSO before IH treatment.

### Cell culture

BV-2 microglial cells and HT-22 neuron cells were obtained from the state Key Laboratory of Brain and Cognitive Science, Chinese Academy of Sciences, which were cultured as described in our previous report [28]. The supernatant medium in microglial cells under both normoxic and IH conditions were used as conditioned medium to coculture HT-22 cells.

### IH cell model

BV-2 cells were placed in a cell incubator with the O_2_ concentration alternation between 1% and 21% within 400 s, in a cyclic repetitive format for 12 h. The normoxia group cells were cultured in a normoxic state (21% O_2_ concentration). After IH treatment, the supernatant medium in both normoxia and IH groups were collected for subsequent experiments.

### SENP1 lentivirus transfection

SENP1 Lentivirus or Lentivirus control solution was added into the medium at a 60%–80% cell density, and the transfection efficiency was then determined.

### Animals

Forty-eight, aged 10-12 weeks, C57BL/6J mice with or without SENP1 knockdown (obtained from the Department of Experimental Animal Science, the School of Medicine, Shanghai Jiao Tong University) were randomly assigned to four groups: SENP1*^+/+^*-Normoxia, SENP1*^+/+^*-IH, SENP1^+/-^-Normoxia and SENP1^+/-^-IH. Experiments were performed complying with the guidelines published by United States National Institutes of Health. The protocol in this study was approved by the animal care and use committee of Xinhua Hospital, Shanghai Jiao Tong University School of Medicine (App. No.: XHEC-F-2019-030). SENP1 knockdown (SENP1^+/-^) mice were generate according to previous study [24]. An equal number of male and female mice were included in each group to avoid any potential sex-related effect. Mice’s genotype identification was conducted before the experiment.

### IH animal model

The normoxia and IH groups mice were placed in two cages (Bio-Instruments, Redfield, NY, USA). IH exposure in mice was achieved by oscillating the O_2_ concentration in the cages via automated, computer-controlled gas exchange systems. The IH protocol consisted of 4-min cycles (2 min of 10% O_2_ and 2 min of 21% O_2_) for 8 h/day during the light period (9 a.m.−5 p.m.). The normoxia group mice were exposed to normoxic condition. For the remaining 16 h, O_2_ concentration was kept at 21% in both IH and normoxia groups. The mice were discontinuously left in cages for 4 weeks and exposed to intermittent hypoxia or normoxic conditions.

### Morris water maze (MWM) test

Cognitive ability, including spatial learning and memory, was assessed by MWM test as previously described [29].

### Animal sample collection

Mice were anesthetized with 5% pentobarbital sodium and then sacrificed to collect the hippocampus or whole brain for subsequent experiments after cardiac saline perfusion.

### qRT-PCR analysis

qRT-PCR was performed as previously described [30]. The primer sequences used in this study are listed: SENP1 forward: AGTAAAGAAGGTTCCGGTTCCCG, reverse: GCCGCCACTCACCGAAC; IL-1β forward: TGCCACCTTTTGACAGTGATG, reverse: AAGGTCCACGGGAAAGACAC; TNF-α forward: GATCGGTCCCCAAAGGGATG, reverse: GGTTTGCTACGACGTGGGC; glyceraldehyde 3-phosphate dehydrogenase (GAPDH) forward: AGGTCGGTGTGAACGGATTTG, reverse: TGTAGACCATGTAGTTGAGGTCA. In brief, total RNA was extracted using RNAiso Plus reagent, reverse-transcribed to complementary DNA, and then amplified using an SYBR-Green master mix kit (Takara). The mRNA levels were calculated relative to GAPDH using the 2^−ΔΔCT^ method.

### Western blot analysis

The total protein of the cells and hippocampus samples was separated by sodium dodecyl sulfate-polyacrylamide gel electrophoresis, transferred onto nitrocellulose membranes, and then incubated with primary antibodies: SENP1 (ab236094, Abcam), SUMO-1 (sc-5308, SANTA), PPARγ (#2443, Cell Signaling Technology), IL-1β (ab234437, Abcam), TNF-α (ab215188, Abcam), NF-κB p65 (ab16502, Abcam), Bcl-2 (ab182858, Abcam), Bax (ab199677, Abcam), Cleaved caspase-3 (ab49822, Abcam) and β-actin (ab179467, Abcam) at 4° overnight, and secondary antibodies: Goat Anti-Rabbit IgG (ab7090, Abcam), Goat Anti-Mouse IgG (ab6789, Abcam) for 1h at room temperature. Protein signals were visualized using the Immobilon Western kit (Millipore, USA). Data were quantitated from at least three independent assays.

### Co-immunoprecipitation (CO-IP) analysis

Cells or fresh hippocampus samples were collected and lysed with ice-cold lysis buffer. After pre-clearing, PPARγ antibody and protein A/G PLUS-agarose were added into the lysate and cultured at 4° C overnight, and the resulting immune complexes were collected for western blot analysis using SUMO-1 and PPARγ antibodies to determine the SUMOylation of protein.

### Cell immunofluorescence analysis

The cells were collected and permeabilized and blocked, incubated with primary SENP1 (ab236094, Abcam) and Alexa Fluor 647 IgG (Ab150115, Abcam) for 1 h. Finally, cell nuclear was stained using 4′,6-diamidino-2-phenylindole (DAPI). Images were captured using a fluorescence microscope.

### ELISA assay

IL-1β and TNF-α in supernatants were measured by ELISA kits (BioLegend, USA) following the manufacturers’ instructions.

### Statistical analysis

Data are presented as mean ± standard deviation (SD). Student's *t* tests, repeated measures analysis of variance (ANOVA), or χ^2^ tests were used to compare the differences among groups. *P* < 0.05 was considered to be significant.

## RESULTS

### IH triggers inflammatory response and neuronal apoptosis

**Figure 1:**
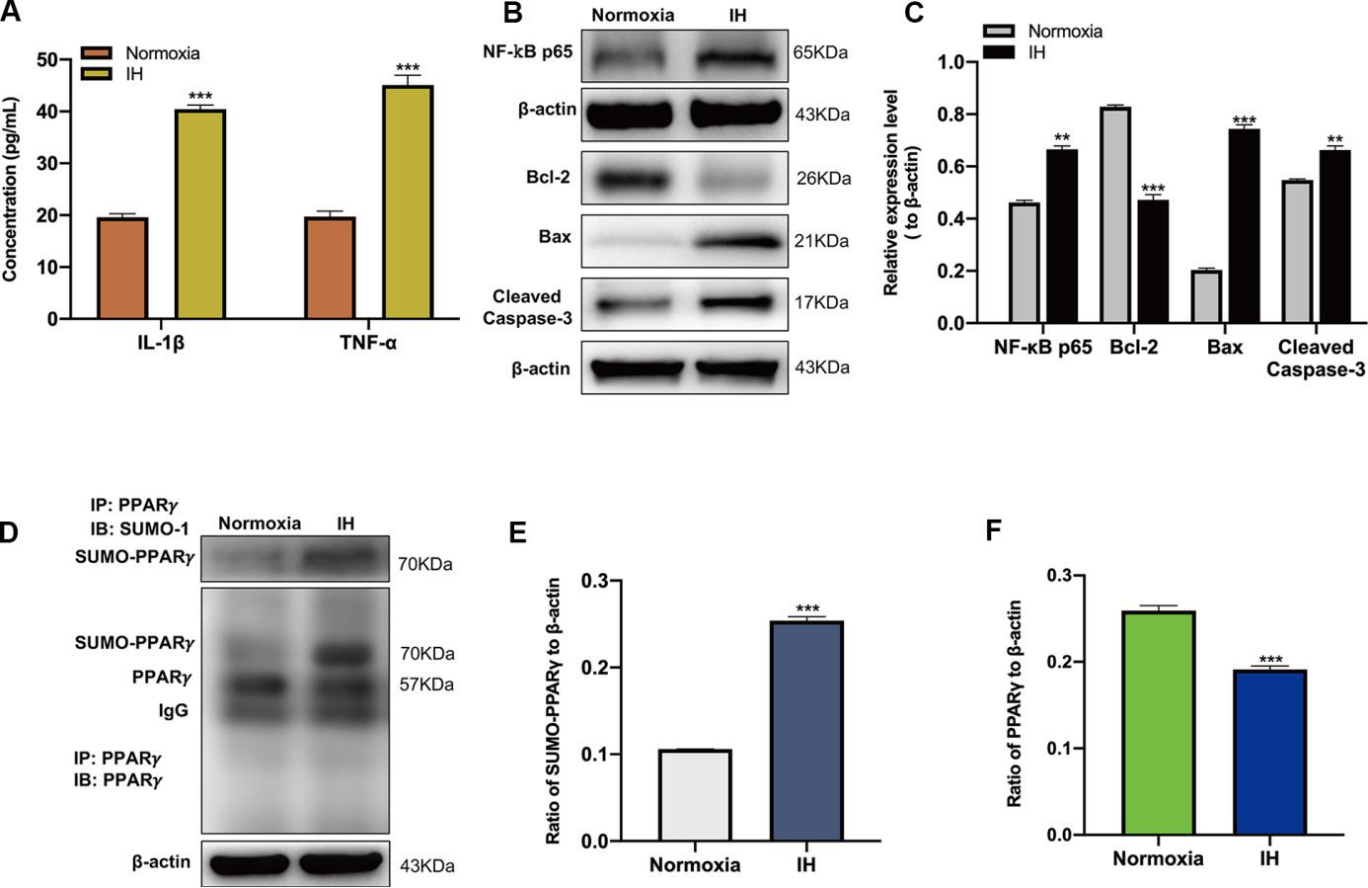
**Intermittent hypoxia (IH) triggers inflammatory response, neuronal apoptosis and the SUMOylation of peroxisome proliferator-activated receptor-γ (PPARγ).** (**A**) The expression of IL-1β and TNF-α in BV-2 cells under normoxic and IH conditions were detected using ELISA. ***p < 0.001 versus the normoxia group. (**B**, **C**) The expression of NF-κB p65 in BV-2 cells and Bcl-2, Bax, Cleaved caspase-3 in HT-22 cells were detected by western blot analysis. **p < 0.01, ***p < 0.001 versus the normoxia group. (**D**, **E**) The effects of IH on the SUMOylation of PPARγ and the level of PPARγ (**D**, **F**) were detected by co-immunoprecipitation followed by western blot analysis. ***p < 0.001 versus the normoxia group. NF-κB p65, nuclear factor kappa B p65. Bcl-2, Bax, Cleaved caspase-3 are apoptosis-related proteins. IL-1β, interleukin-1β; TNF-α, tumor necrosis factor-α. Normoxia: BV-2 cells were cultured in normoxic condition. IH: BV-2 cells were exposed to IH. SUMO, small ubiquitin-like modifier; ELISA, enzyme-linked immunosorbent assay; IP, immunoprecipitation; IB, immunoblot.

After IH treatment, the expression of IL-1β, TNF-α and NF-κB p65 were detected using ELISA and western blot analysis. Results showed that the expression of IL-1β, TNF-α and NF-κB p65 were significantly increased in comparison with that in BV-2 cells with normoxic treatment (Figure 1A–1C), indicating that IH induced an inflammatory response *in vitro*. After the coculture of HT-22 cells with conditioned medium, which collected from the cultured BV-2 cells under normoxic and IH conditions, and the apoptosis-related proteins were detected by western blot analysis. Results showed that the expression of Bax, Cleaved caspase-3 were significantly increased, and the expression of Bcl-2 was significantly decreased compared to that cocultured with normoxic conditioned medium (Figure 1B, 1C), suggesting that the inflammatory responses induced by IH could lead to neuronal apoptosis.

### Depletion of SENP1 promotes the IH-induced inflammatory response and neuronal apoptosis by increasing the SUMOylation of PPARγ

**Figure 2:**
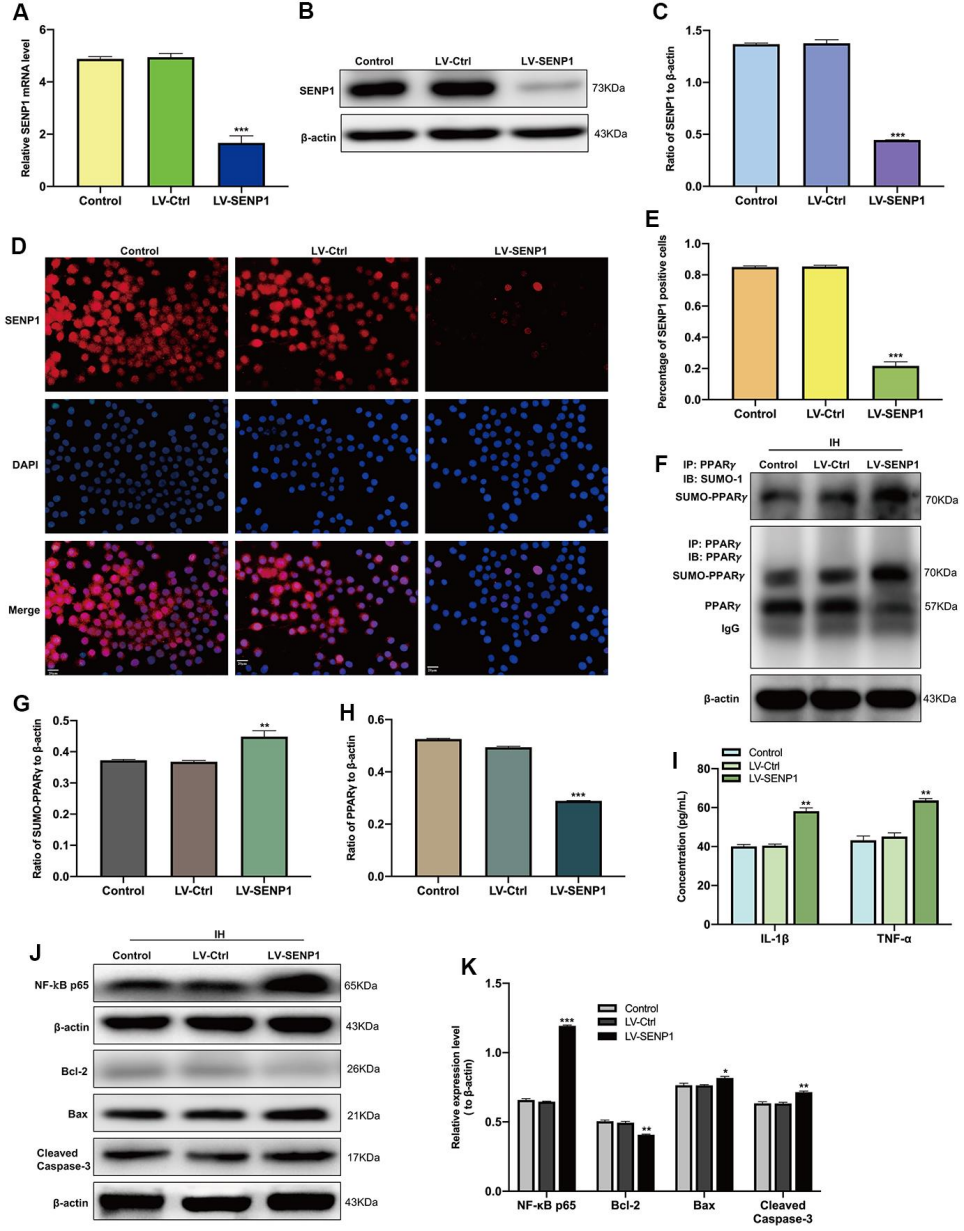
**SENP1 depletion promotes the SUMOylation of peroxisome proliferator-activated receptor-γ (PPARγ) in BV-2 cells and neuronal apoptosis under intermittent hypoxia (IH) condition.** (**A**) The qRT-PCR, (**B**, **C**) western blot analysis, and (**D**) cellular immunofluorescence analysis showed the downregulation of SENP1 in BV-2 cells after transfection. Untreated BV-2 cells were used as controls. Scale bar, 20 μm. ***p < 0.001 versus LV-Ctrl. (**E**) Data are presented as the percentage of SENP1 positive BV-2 cells. ***p < 0.001 versus LV-Ctrl. (**F**–**H**) The effects of IH on the SUMOylation of PPARγ (**F**, **G**) and the level of PPARγ (**F**, **H**) were detected by co-immunoprecipitation followed by western blot analysis. **p < 0.01, ***p < 0.001 versus LV-Ctrl. (**I**) The expression of IL-1β and TNF-α in BV-2 cells with or without SENP1 knockdown under IH condition were detected using ELISA. ***p < 0.001 versus LV-Ctrl. (**J**, **K**) The expression of NF-κB p65 in BV-2 cells and Bcl-2, Bax, Cleaved caspase-3 in HT-22 cells were detected by western blot analysis. *p < 0.05, **p < 0.01, ***p < 0.001 versus LV-Ctrl or HT-22 cocultured with the medium of LV-Ctrl BV-2 cells under IH condition. SENP1, small ubiquitin-related modifier protein-specific protease 1; LV, Lentivirus; qRT-PCR, quantitative reverse transcriptase polymerase chain reaction.

The SUMOylation of PPARγ was then estimated by CO-IP analysis, which showed that IH upregulated the SUMOylation of PPARγ and decreased the level of PPARγ in BV-2 cells (Figure 1D–1F). To identify the regulatory mechanism of SENP1 on the IH-induced inflammatory response, we downregulated the expression of SENP1 in BV-2 cells and then examined the levels of proteins associated with inflammation and apoptosis in BV-2 cells or HT-22 cells. After transfection, the expression of SENP1 was significantly downregulated, which was estimated by qRT-PCR (Figure 2A), western blot analysis (Figure 2B, 2C), and cellular immunofluorescence analysis (Figure 2D, 2E). Subsequently, we evaluated the effect of SENP1 on the SUMOylation of PPARγ using CO-IP analysis under IH condition, which showed that SENP1 depletion significantly enhanced the SUMOylation of PPARγ (Figure 2F, 2G) and further reduced the level of PPARγ (Figure 2F, 2H) in comparison with that in BV-2 cells without SENP1 knockdown. To determine the effect of SENP1 depletion on inflammatory responses and neuronal apoptosis, the expression of IL-1β, TNF-α and NF-κB p65 were detected using ELISA and western blot analysis, which showed that the expression of IL-1β, TNF-α and NF-κB p65 were significantly increased in comparison with that in BV-2 cells with SENP1 Knockdown (Figure 2I–2K), indicating that SENP1 depletion promoted inflammatory response induced by IH. The apoptosis-related proteins in HT-22 cells then were detected by western blot analysis, which showed that SENP1 depletion upregulated the expression of Bax, Cleaved caspase-3 and downregulated the expression of Bcl-2 compared to that cocultured with conditioned medium in BV-2 cells without SENP1 Knockdown (Figure 2I–2K), suggesting that the inflammatory response promoted by SENP1 depletion could enhance neuronal apoptosis under IH condition.

**Figure 3:**
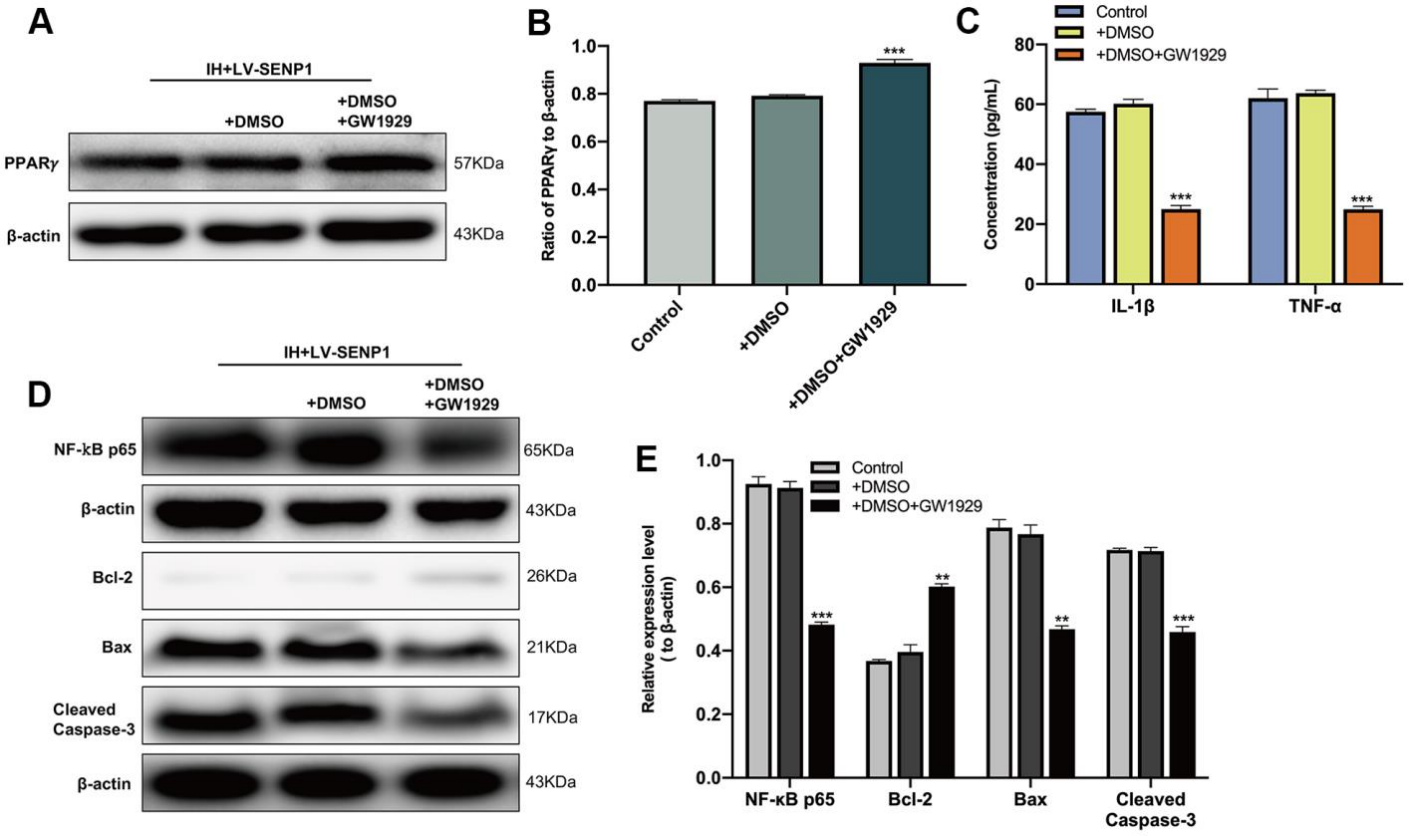
**The effect of peroxisome proliferator-activated receptor-γ (PPARγ) on the inflammatory response and neuronal apoptosis under intermittent hypoxia (IH) condition.** (**A**, **B**) The expression of PPARγ in BV-2 cells with SENP1 knockdown under IH condition was detected by western blot analysis. ***p < 0.001 versus the dimethyl sulfoxide (DMSO) group. The DMSO group, BV-2 cells were pretreated with 0.1% DMSO; DMSO+GW1929 group, BV-2 cells were pretreated with 10 μM GW1929 and 0.1% DMSO; untreated BV-2 cells with SENP1 knockdown were used as the control group. (**C**) The expression of IL-1β and TNF-α in BV-2 cells with SENP1 knockdown under IH condition were detected using ELISA. ***p < 0.001 versus the DMSO group. (**D**, **E**) The expression of NF-κB p65 in BV-2 cells and Bcl-2, Bax, Cleaved caspase-3 in HT-22 cells were detected by western blot analysis. **p < 0.01, ***p < 0.001 versus the DMSO group or HT-22 cocultured with the medium of LV-SENP1 BV-2 cells, which added DMSO under IH condition. GW1929, a potent PPARγ agonist.

To determine the effect of PPARγ on the IH-induced inflammatory response and neuronal apoptosis, the reduction of PPARγ in SENP1 knockdown BV-2 cells was interrupted by the PPARγ agonist, GW1929. Western blot analysis (Figure 3A, 3B) showed that the expression of PPARγ was significantly upregulated than that in the dimethyl sulfoxide (DMSO) group, and no significant difference between the DMSO group and the control group. The protein expression of IL-1β, TNF-α and NF-κB p65 were then detected by ELISA and western blot analysis, which showed that the administration of GW1929 significantly inhibited the expression of IL-1β, TNF-α and NF-κB p65 (Figure 3C–3E). The apoptosis-related proteins in HT-22 cells then were detected by western blot analysis, which showed that the upregulation of PPARγ decreased the protein expression of Bax, Cleaved caspase-3 and increased the expression of Bcl-2 compared with the DMSO group, and there was no significant difference between the DMSO group and group (Figure 3D, 3E), suggesting that the inflammatory responses and neuronal apoptosis promoted by SENP1 depletion under IH condition could abolished by the application of GW1929.Together, these results suggested that SENP1 depletion promoted the IH-induced inflammatory response and neuronal apoptosis by accelerating the SUMOylation of PPARγ in BV-2 cells.

### Depletion of SENP1 promotes the SUMOylation of PPARγ induced by IH in hippocampus

**Figure 4:**
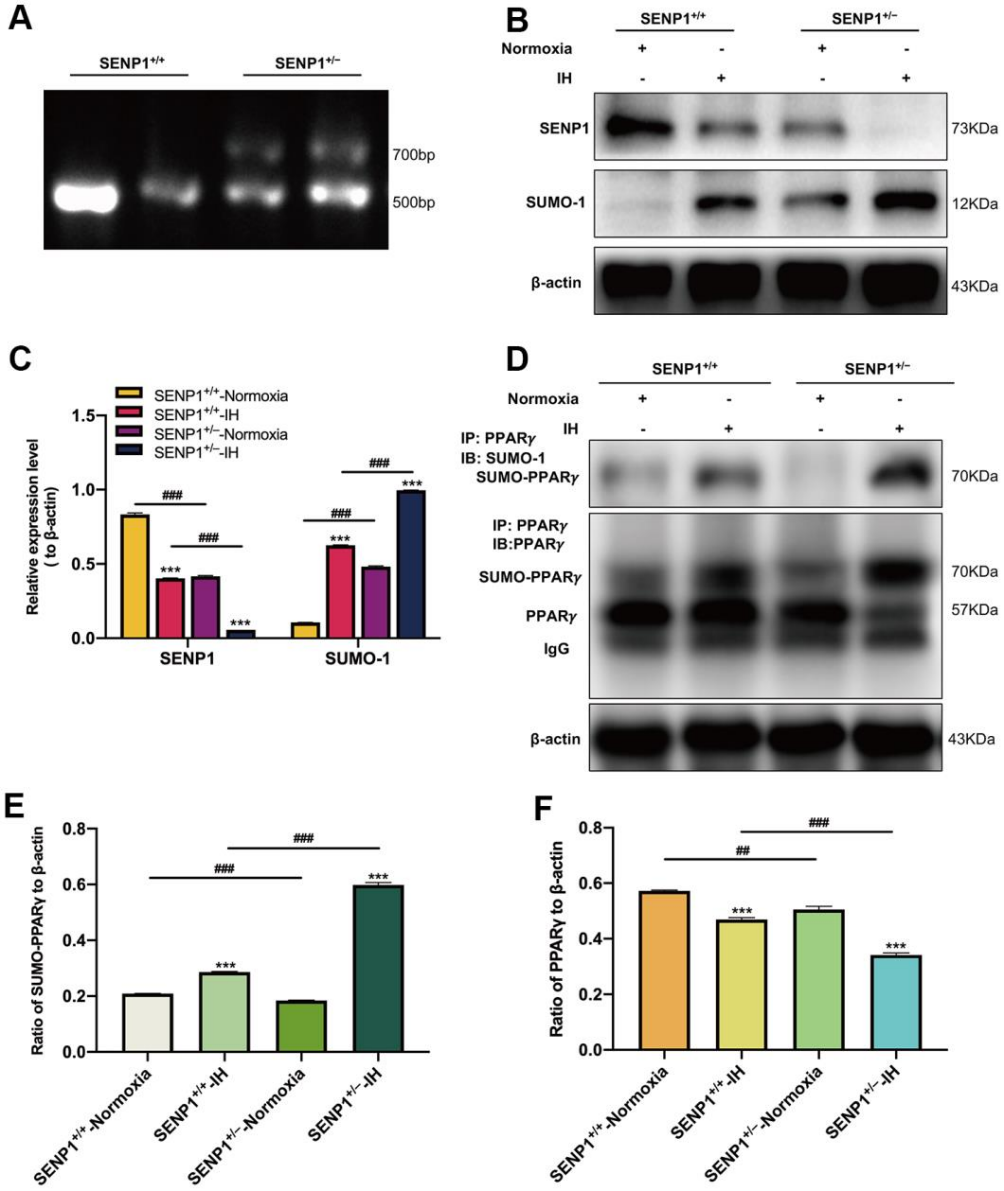
**SENP1 depletion promotes the SUMOylation of peroxisome proliferator-activated receptor-γ (PPARγ) in mice under intermittent hypoxia (IH) condition.** (**A**) The genotype of mice was identified by agarose gel electrophoresis. (**B**, **C**) The expression of SENP1 and SUMO-1 in mice with or without SENP1 knockdown under normoxic and IH conditions. ***p < 0.001 versus the normoxia groups; ^###^*p* < 0.001 versus the SENP1^+/+^-IH group. (**D**–**F**) The effects of IH on the SUMOylation of PPARγ (**D**, **E**) and the level of PPARγ (**D**, **F**) were detected by co-immunoprecipitation followed by western blot analysis. ***p < 0.001 versus the normoxia groups. ^##^*p* < 0.01, ^###^*p* < 0.001 versus the SENP1^+/+^-IH group. Normoxia: mice were treated under normoxic condition. IH: mice were exposed under IH. SENP1^+/+^, wild type mice; SENP1^+/−^, SENP1 knockdown mice.

To investigate the effect of SENP1 depletion on the SUMOylation of PPARγ *in vivo*, we knocked down the gene expression of SENP1 in mice using gene-editing technology. The genotype of mice and the knockdown efficiency of SENP1 were identified using DNA agarose electrophoresis and western blot analysis, respectively. DNA agarose electrophoresis results showed that the genotype of wild-type mice was that of a homozygote, and the SENP1 knockdown mice’s genotype was that of a mutant heterozygote (Figure 4A). The knockdown efficiency results showed that the expression of SENP1 was significantly decreased in SENP1 knockdown mice compared with that in wild-type mice (Figure 4B, 4C). In addition, we also observed that the expression of SENP1 was decreased in both IH groups compared with that in normoxia groups, and it was lower in the SENP1 knockdown group than that in the wild-type group under IH condition. In contrast, the expression of SUMO-1 in hippocampus of mice was significantly increased after SENP1 knockdown under IH and normoxic conditions, and IH further increased this enhancement (Figure 4B, 4C). These results suggested that SENP1 knockdown enhanced the SUMOylation *in vivo*. After IH treatment, the effect of SENP1 depletion on the SUMOylation of PPARγ in hippocampus of mice with or without SENP1 knockdown was evaluated using CO-IP, which showed that IH promoted the SUMOylation of PPARγ, and significantly decreased the level of PPARγ. Moreover, SENP1 depletion significantly promoted these alterations (Figure 4D–4F), suggesting that SENP1 depletion downregulated the level of PPARγ in hippocampus of mice by accelerating the IH-induced SUMOylation of PPARγ.

### Depletion of SENP1 promotes the inflammatory response and neuronal apoptosis induced by IH in hippocampus

**Figure 5:**
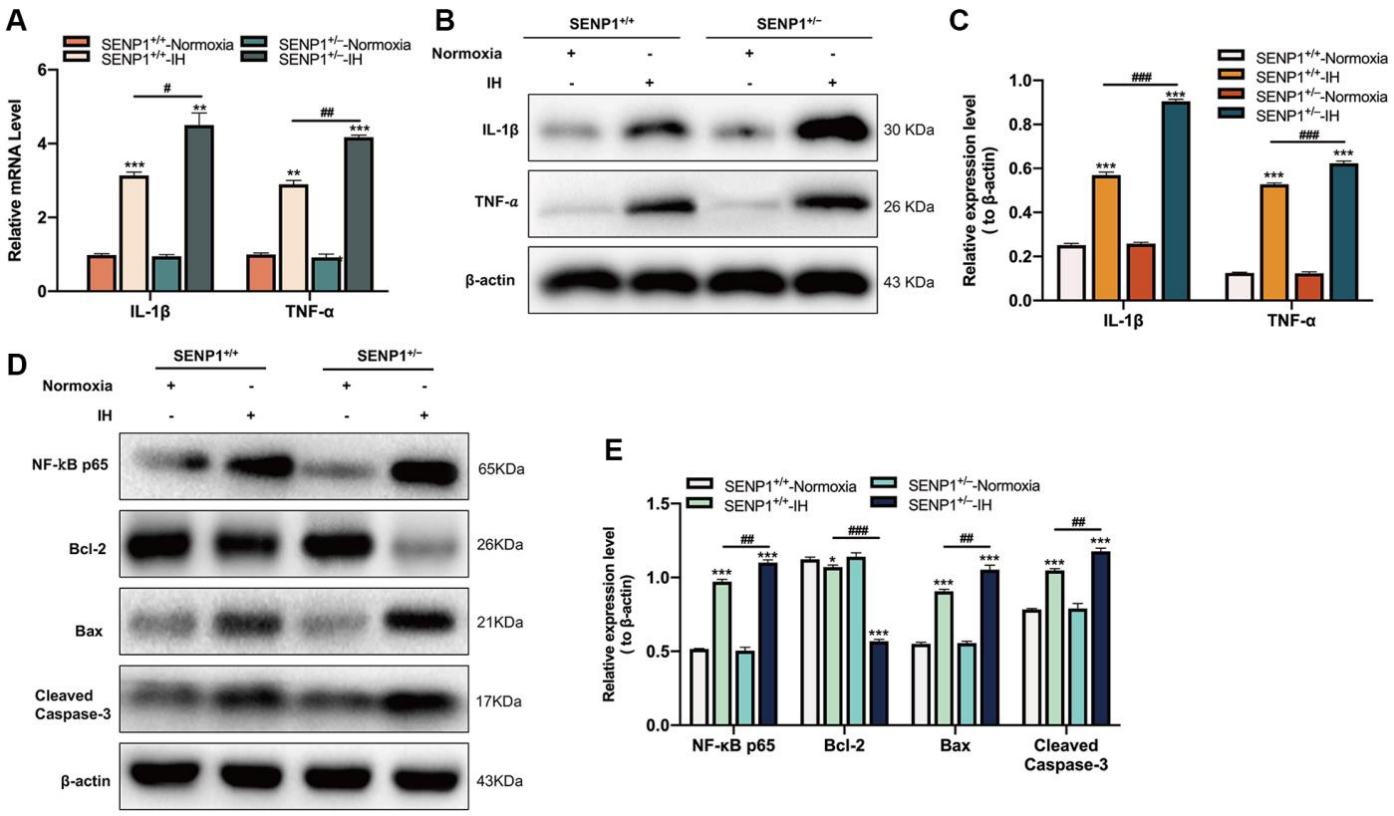
**SENP1 depletion promotes the inflammatory response and neuronal apoptosis under intermittent hypoxia (IH) condition.** (**A**–**C**) The expression of IL-1β and TNF-α in hippocampus under normoxic and IH conditions were detected by (**A**) qRT-PCR and (**B**, **C**) western blot analysis. **p < 0.01, ***p < 0.001 versus the normoxia group. ^#^*p* < 0.05, ^##^*p* < 0.01, ^###^*p* < 0.001 versus the SENP1^+/+^-IH group. (**D**, **E**) The expression of NF-κB p65, Bcl-2, Bax, Cleaved caspase-3 in hippocampus were detected by western blot analysis. *p < 0.05, ***p < 0.001 versus the normoxia group. ^##^*p* < 0.01, ^###^*p* < 0.001 versus the SENP1^+/+^-IH group.

Subsequently, the expression of IL-1β and TNF-α in hippocampus of mice were evaluated by qRT-PCR and western blot analysis. qRT-PCR results showed that IH significantly upregulated the expression of IL-1β and TNF-α, and the upregulation in SENP1 knockdown mice was more obvious than that in wild-type mice (Figure 5A). Western blot analysis results showed a similar trend: IH increased the inflammatory response, and SENP1 depletion further promoted the enhancement induced by IH (Figure 5B, 5C). The NF-κB p65 and apoptosis-related proteins in hippocampus of mice were detected by western blot analysis, which showed that IH significantly increased the expression of NF-κB p65, Bax, Cleaved caspase-3 and decreased the expression of Bcl-2 compared to that with normoxic treatment, and the enhancement in SENP1 knockdown mice was more obvious than that in wild-type mice (Figure 5D, 5E), suggesting that SENP1 depletion promoted the inflammatory response and neuronal apoptosis induced by IH in hippocampus of mice.

### Depletion of SENP1 promotes IH-induced cognitive dysfunction

**Figure 6:**
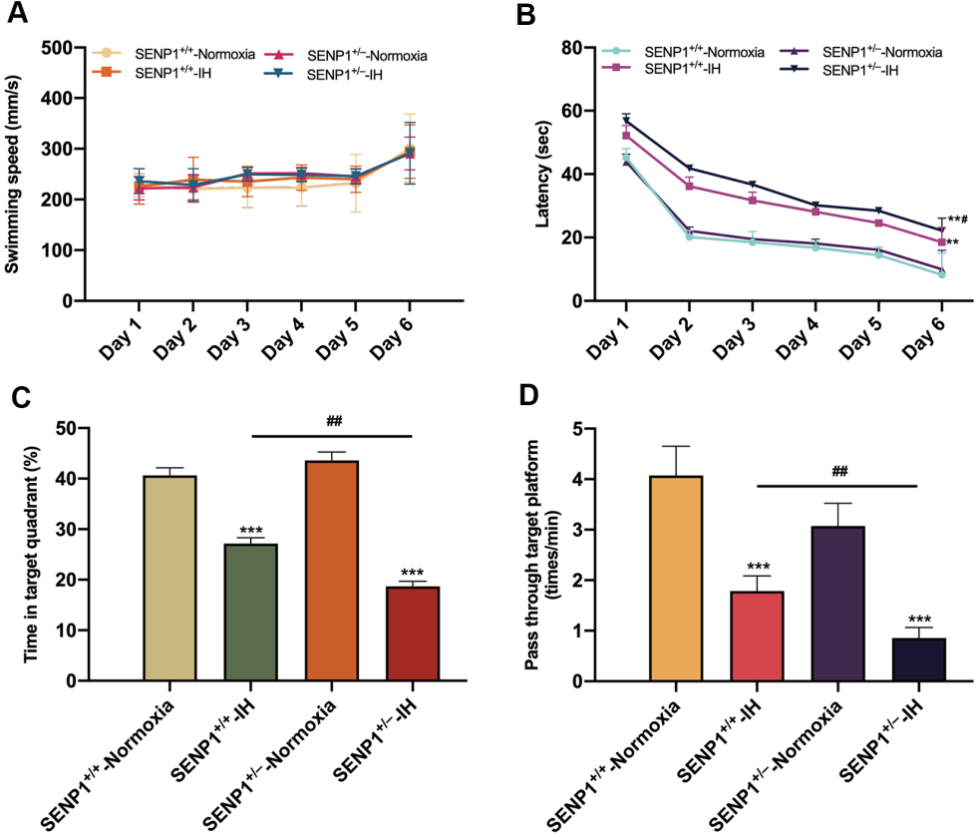
**SENP1 depletion promotes intermittent hypoxia (IH)-induced cognitive decline.** (**A**–**D**) Cognitive functions of mice with or without SENP1 knockdown under normoxic and IH conditions were analyzed using the Morris water maze test. Mice swam in the pool for 60 s on day 6, and other days’ test termination times were subject to the climbing platform. (**A**) There was no difference among the four experimental groups in average swimming speed throughout six consecutive days. (**B**) Latency was the time that mice first climbed onto the hidden platform or first passed through the area where the platform was located. (**C**) The percentage of time that mice spent in the quadrant where the platform was located versus the total time. (**D**) The frequency of passing through the area where the platform was located. **p < 0.01, ***p < 0.001 versus the normoxia groups. ^##^*p* < 0.01 versus the SENP1^+/+^-IH group.

Before IH treatment, the learning and memory abilities of spatial orientation in mice were evaluated by the MWM test, which showed that mice’s learning and memory abilities of spatial orientation and swimming speed were not significantly different (*p* > 0.05, Supplementary Figure 1), indicating that SENP1 depletion did not affect mice’s cognitive ability and motor ability under normoxic condition. After IH treatment, MWM test results showed that mice’s swimming speed had no statistical difference throughout six consecutive days (*p* > 0.05, Figure 6A), suggesting that neither IH nor SENP1 depletion affected the motor ability of mice. However, both IH groups exhibited longer escape latencies than the normoxia groups, and the SENP1 knockdown group exhibited a much longer latency than that in the wild-type group on day 6 (Figure 6B). In addition, the time percentage in the target quadrant and the frequency passing through the platform area were significantly reduced in both IH groups. Notably, the reductions were more obvious in the SENP1 knockdown group (Figure 6C, 6D). Together, these results suggested that IH impaired the cognitive ability in mice, and SENP1 depletion further deteriorated this impairment.

## DISCUSSION

The findings in the present study are: (1) IH induced cognitive impairment in mice, which was aggravated by SENP1 depletion; (2) IH led to the inflammatory response and neuronal apoptosis *in vitro* and *in vivo*, which were further enhanced by SENP1 depletion; (3) SENP1 depletion may down-regulated the expression of PPARγ in microglia and hippocampus by promoting the SUMOylation of PPARγ induced by IH; and (4) SENP1 depletion aggravated the microglia-mediated inflammatory response and neuronal apoptosis, thus exaggerating IH-induced cognitive decline, probably by promoting the SUMOylation of PPARγ. These results collectively suggested that inflammation in hippocampus played a crucial role in IH-associated cognitive decline, and SENP1 depletion aggravated microglia-mediated neuroinflammation and neuronal apoptosis in hippocampus by promoting the SUMOylation enhancement of PPARγ, and contributing to the exaggeration of cognitive decline.

Microglia-mediated inflammation plays a crucial role in the development and the repair in brain [31, 32], and is also associated with IH-related cognitive dysfunction [1]. Activated microglia secrete IL-1β, TNF-α, adhesion molecules and other signaling mediators [33]. The cytokines can destroy the blood-brain barrier and activate much more microglial cells, leading to a cycle of malignant inflammation [4]. Persistent increase of pro-inflammatory cytokines and microglial activation may cause a chronic inflammatory status leading to neuronal damage [34, 35]. Microglia can be activated as M1 and M2 phenotypes. The M1 phenotype predominates at the neuroinflammation site and is related to the secretion of proinflammatory cytokines and chemokines. In contrast, the M2 phenotype appears later and performs tissue repair via the secretion of anti-inflammatory cytokines [36, 37]. Previous studies have shown that hypoxia might facilitate M1 phenotype polarization and attenuate M2 phenotype activation [38]. Therefore, considering the critical role of neuroinflammation in neurological damage induced by IH, the regulation of M1 and M2 phenotype microglia in neuroinflammation of is an essential factor in the damage of CNS. Although microglia have been recognized as an important contributor to IH-induced neuroinflammation, the activating mechanism remains unclear. Among the rare studies, a study found that the microglia-mediated neuroinflammation might directly impact CNS damage through the TLR4 pathway and the release of pro-inflammatory cytokines under IH condition [39]. Our previous study also confirmed that IH could trigger the M1 phenotype of microglia, suggesting that the crucial role of microglia in neurological injury induced by IH [40]. Therefore, as shown in the present study, the significant upregulation of IL-1β, TNF-α and NF-κB p65 expression in IH treated microglia indicated that IH induced the inflammatory response in microglia by skewing to M1 polarization.

SUMOylation is a reversible process, which is catalyzed by SUMO-specific enzymes [27]. The de-SUMOylation is conducted by a SUMO-specific proteases (SENPs) family. SENP1 was the first identified SUMO-specific protease among the six SENPs, which is widely located in cell nucleus and participates in various diseases [11, 22, 23]. SENP1 depletion destroyed the growth, migration and invasion in hepatoma carcinoma cell *in vitro* and *in vivo* [25]. Besides, SENP1 depletion in adipocytes elevated the activity of NF-κB, the production of cytokines and pancreatitis [11]. Our previous study revealed that SENP1 overexpression suppressed the IH-induced inflammatory response in microglia [28]. In the present study, SENP1 depletion significantly promoted the IH-induced SUMOylation enhancement of PPARγ and reduced the level of PPARγ *in vitro* and *in vivo*, thus contributing to the enhancement of neuroinflammation and neuronal apoptosis. After the administration of the PPARγ agonist, GW1929, the enhancement of neuroinflammatory response and neuronal apoptosis promoted by SENP1 depletion after IH treatment was abolished. Collectively, SENP1 depletion may synchronously decrease the activation of PPARγ and increase the inflammation in microglia through promoting the SUMOylation of PPARγ induced by IH, thus enhancing neuronal apoptosis.

PPARγ is expressed in microglia, astrocyte and neuronal cells, and plays a crucial role in inflammatory response. Activated PPARγ performs a significant anti-inflammatory effect by inhibiting the activation of NF-κB pathway and the production of proinflammatory cytokines [41, 42]. Compelling evidences suggested that the activation of PPARγ alleviated neuroinflammation related to chronic neurological insults [41, 43], the inhibition of PPARγ activity exacerbated inflammatory response in brain and peripheral tissues insults [44–46]. A recent study demonstrated that the downregulation of PPARγ is associated with the neuroinflammation in hippocampus and cognitive decline in mice [47]. To explore the possible regulatory mechanism underlying SENP1 on neuroinflammation, after downregulated in the expression of SENP1, we next examined the SUMOylation and the expression of PPARγ under normoxic and IH conditions *in vitro* and *in vivo*. In accord with previous reports, our results exhibited that IH induced the SUMOylation of PPARγ and reduced the expression of PPARγ in both IH groups. In addition, SENP1 depletion further promoted the SUMOylation of PPARγ and the reduction of PPARγ after IH treatment. Notably, SENP1 depletion did not cause the SUMOylation enhancement of PPARγ, the decrease of PPARγ, and neuroinflammation in mice under normoxic condition, but it led to the aggravated neuroinflammation in IH-treated mice. These observations suggested that SENP1 depletion alone induced the SUMOylation enhancement without any influence on the SUMOylation of PPARγ, and may be insufficient to cause the reduction of PPARγ. However, enhanced SUMOylation may enhance the sensitivity to IH insult, lead to the decrease of PPARγ. In addition, we also found that IH-induced neuroinflammation led to neuronal apoptosis and neuroinflammation that aggravated by SENP1 depletion promoted the apoptosis, thus contributing to the consequential exaggeration of cognitive decline. These findings also indirectly indicated that the de-SUMOylation of PPARγ played a protective role in IH impairment. Notably, PPARγ may also have protection from oxidative stress by upregulating antioxidant and copper-zinc superoxide dismutase [41]. Previous study revealed that PPARγ depletion could promote oxidative stress injury in brain [48], and IH could induce oxidative stress in hippocampus, resulting in mice’s cognitive deficits [49]. We cannot exclude the possibility that IH-induced the decrease of PPARγ in hippocampus through other means, such as increasing oxidative stress response, which led to the cognitive decline. Additionally, the destruction of structural integrity in hippocampus is crucial to the severity and development of neurodegenerative disorders related to cognitive defect [50]. It has been proved that PPARγ downregulation can induce neuronal apoptosis, destroy neurogenesis and synaptic plasticity, thus resulting in the damage of learning and memory ability in mice [51]. Thus, the alteration of structural integrity in hippocampus may be another possible mechanism underlying this study’s cognitive impairment.

There are also shortcomings in this study. Firstly, PPARγ is also expressed in astrocytes and other cells involved in the CNS inflammatory response. PPARγ in the astrocytes has been demonstrated to participate in neuroinflammatory response [41]. However, astrocytes were not investigated in the present study, which are furtherly needed to examine whether astrocytes participate in the neuroinflammatory response and cognitive impairment induced by IH. Secondly, the regulatory mechanism of PPARγ on NF-κB pathway in IH-induced neuroinflammation was not investigated in the study.

In summary, this study demonstrated that microglia-mediated inflammatory response played essential roles in neuronal apoptosis and IH-associated cognitive decline, and that SENP1 depletion exaggerated microglia-mediated inflammation and subsequently neuronal apoptosis probably by promoting the SUMOylation of PPARγ, thus contributing to the downregulation of PPARγ in hippocampus and IH-related cognitive decline of mice. These findings may, in the future, provide clinical choices for patients with OSA.

### Data availability statement

The data used to support the findings of this study are included in the article.

## Supplementary Material

Supplementary Figure 1
