# Supplementary material for: Depletion of SENP1-mediated PPARγ SUMOylation exaggerates intermittent hypoxia-induced cognitive decline by aggravating microglia-mediated neuroinflammation
Source: Aging (Albany NY). 2021 May 25;13(11):15240–54. doi: 10.18632/aging.203084 (PMC8221356; doi:10.18632/aging.203084)
Supplement: Supplementary Figure 1 [file aging-13-203084-s001.pdf]

SUPPLEMENTARY FIGURE

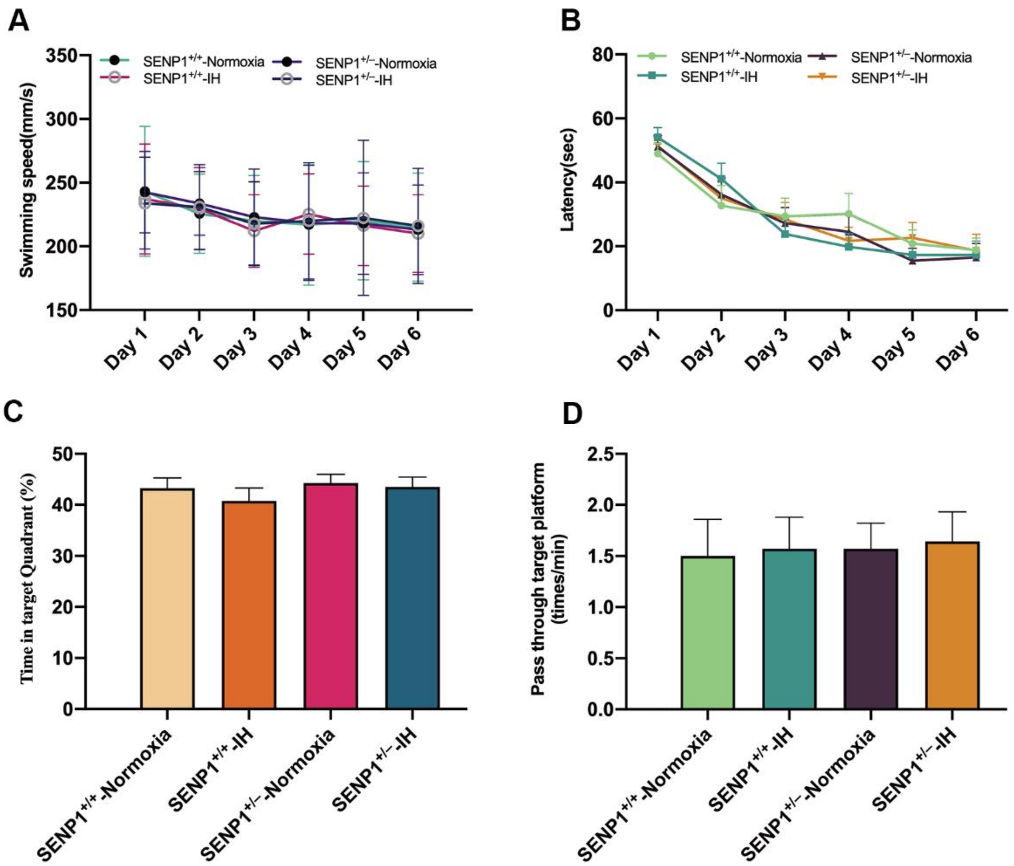

Supplementary Figure 1. Assessment of spatial learning and memory with Morris Water Maze (MWM) test before the intermittent hypoxia (IH) treatment. (**A**) Swimming speed throughout 6 consecutive days among four groups. (**B**) Latency, the time to find the hidden platform. The platform was removed at day 6, and the latency is the first time to enter the platform area. (**C**) After the removal of platform, the time percentage of mice spent in the quadrant platform area in 60 s. (**D**) Times of mice passing through target platform. Data are expressed as mean ± SD (n = 12 per group). SENP1$^{+/+}$, represents the wild-type mice. SENP1$^{+/-}$, represents SENP1 knockdown.
